# The effect of ramet mortality on clonal plant growth

**DOI:** 10.1007/s12064-019-00274-6

**Published:** 2019-02-08

**Authors:** Veronika Benedek, Péter Englert

**Affiliations:** Department of Plant Taxonomy, Ecology and Theoretical Biology, ELTE Eötvös Loránd University, Pázmány Péter Stny. 1/C, Budapest, Hungary

**Keywords:** Clonal plant, Ramet mortality, Cellular automaton, Population dynamics

## Abstract

Clonal plants grow horizontally by producing multiple physiological individuals (ramets). We studied clonal growth in a homogeneous environment using a dynamic spatial model based on a stochastic cellular automaton. We investigated different growth forms from the aspect of ramet mortality. Non-steady-state and quasi-steady-state cases were defined, and we determined the number of steps suitable for making a reliable difference between these two types of cases. This given number of steps was used when testing for the proportion of quasi-steady-state cases in 1000 repetitions. We also tested the efficiency of occupation in these cases. Our expectation was that higher occupation would be associated with lower ramet mortality. The results only partially verified this hypothesis. Though with increasing ramet mortality, the average number of ramets tended to decrease, it was not the lowest ramet mortality that resulted in the highest occupation. Our results showed that very low ramet mortality was unfavourable for the plant, as the spreading front and the area behind this front were so packed that the plant was not able to return and recolonize the vacated sites in the central area. This resulted in a lower proportion of quasi-steady-state cases and lower occupation in these cases. Our results may contribute to a deeper understanding of clonal plant growth and its limiting factors.

## Introduction

In clonal plants, a whole plant developed from a single zygote is referred to as a “genet”, and it often consists of a number of clonally formed offspring, referred to “ramets”. This terminology was coined by Kays and Harper (1974). Ramets (“feeding sites”) are interconnected by spacers (Bell 1984) such as stolons or rhizome segments, which can be important for the placement of ramets and the storage and transport of resources (Dong and de Kroon 1994).

A newly established ramet is either able to become physiologically independent or to maintain its between-ramet connections. Clonal integration, the ability to transfer water and nutrients through the connections, has several advantages for clonal plants, e.g. it allows them to cope with environmental heterogeneity by alleviating local resource shortages, influencing competitive ability, increasing invasiveness and altering the species composition and invasibility at the community level (Liu et al. 2016). The degree of this physiological integration has a great variability between species, but two extreme strategies can be distinguished: genet splitters (established ramets gain complete physiological independence) versus integrators (preserve between-ramet connections throughout the lifespan of ramets) (see Oborny et al. 2000). An integrated clone can buffer itself against the spatio-temporal heterogeneity of the habitat, but according to the simulation study by Kun and Oborny (2003), this buffering is not necessarily advantageous in all habitat types. Furthermore, the effect of resource translocation between ramets strongly interacts with the architectural rules of the plant growth: plants with ramets staying alive when a spacer is formed are much less sensitive to change in translocation than plants with ramets only at the tip. If translocation cost is low, translocating plants are better competitors in most cases than plants that do not translocate (Herben 2004).

Each ramet has the potential to perform all biological functions necessary for the survival and reproduction of a whole plant. In this respect, a fully established ramet can be regarded as an individual (Liu et al. 2016). Each ramet has its own birth and mortality event. Several studies suggested considering a clonal plant as a population of ramets (e.g. White 1979; Sackville Hamilton et al. 1987). The birth and mortality rates of ramets influence the size and fitness of genets (Hartnett and Bazzaz 1985).

Birth rate and ramet longevity (i.e. the lifespan of an individual ramet) show a considerable variability between species. A community analysis on meadow plant species found the branching intensity of min. 1, max. 11 rhizome branches per ramet per year and revealed three major categories according to ramet longevity: species with annual ramets, species with perennial ramets and species with mostly biennial ramets (Tamm et al. 2002). Death of ramets may also be caused by pests, e.g. in water hyacinth, *Eichhornia crassipes* (Mart.) Solms, where ramets are virtually immortal (complete tissue replacement occurs every few months), but destruction of the apical meristem results in the death of the ramet via loss of regenerative capability (Center 1987). Also, there are scarce examples for density-dependent ramet mortality, notedly shoot self-thinning in clonal plants (de Kroon and Kalliola 1995). “Yoda’s power law” predicts an increase in average plant biomass associated with a decrease in plant density for a crowded even-aged plant population. The log–log plot of average plant mass versus plant density reveals a straight “self-thinning” line of slope − 3/2 (e.g. Weller 1987). In populations of clonal perennial herbs, however, ramet density is usually restricted by environmental controls and certain rules internal to the plant (Hutchings 1979).

Ramet mortality plays a role in the emergence of some interesting real-world spatial patterns, e.g. central die-back and fairy rings. During central die-back, the oldest spacers and storage organs at the centre of a clonal patch die, leaving an empty central area. Spacers may grow back into this area, leaving a “dead zone” between the re-populated region and the previously existing patch. This annual dead zone is known as a “fairy ring” (Wong et al. 2011). Many clonal plants are characterized by tussock growth forms. In a simulation study, Herben and Novoplansky (2008) examined self-/non-self-discrimination on the spatial distribution and patterning of ramets. They showed that compact tussocks can be generated as a transient phenomenon that typically disappears at equilibrium, when space is uniformly populated by intermingled ramets of different genets, and this outcome is reached independently of architectural and growth attributes of the plants.

We investigated the effects of ramet mortality on clonal growth by means of a model, based on a cellular automaton. This model provides a convenient tool for investigating several ramet generations in several repetitions. We tested, *inter alia*, the efficiency of occupation (i.e. number of ramets in the field) at different ramet mortality rates. Our expectation was that higher ramet mortality would be associated with lower occupation.

## Methods

We investigated clonal growth in a homogeneous environment using a stochastic, spatially explicit dynamic model (see Oborny and Englert 2012). The space was represented by a two-dimensional lattice consisting of 150 × 150 hexagonal cells, called “sites”. The boundaries were closed.

**Fig. 1:**
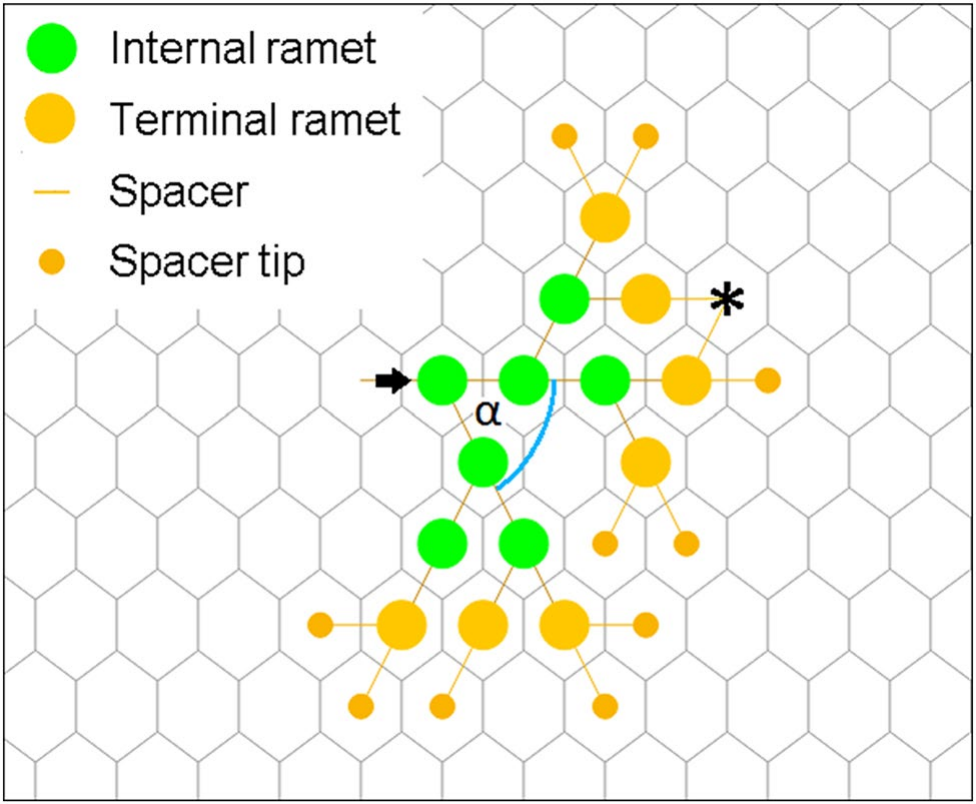
Growing clonal plant architecture in the hexagonal lattice (seventh step of growing). The plant consists of internal and terminal ramets, spacers and spacer tips. *α* is the branching angle, i.e. the angle of divergence from the principal direction of growth. The lattice size in our simulations was larger than in this example. Marked with asterisk when two branches try to produce spacer tips into the same cell; one of the spacer tips will be terminated randomly, see updating rules below

Every simulation instance represented the growth of a single clone (genet), which consisted of ramets and spacers between ramets. The process started from one seed placed into the otherwise empty field at a randomly selected site near the centre of the field with a pre-defined principal direction of growing. In the first step, the seed developed into a ramet and produced two spacer tips into adjacent cells, one in the principal growing direction of the branch and another laterally, with an α branching angle (see Fig. 1), to the left or to the right with equal probability (*P* = 0.5). In the second step, as each site could contain only a single ramet (i.e. could be empty or occupied), new ramets were produced only if the chosen adjacent sites were empty. In the third step, they initiated new spacers. If a chosen site was occupied or the stolon tip hits the boundary, the affected stolon tip got aborted.

Only those ramets could produce new ramets that were born in the previous generation. They were called “terminal ramets”, as they were in terminal positions along the branches. Every terminal ramet tried to develop two new ramets into adjacent cells. All the remaining ramets, i.e. ramets in internal positions, were called “internal ramets”. Internal ramets were inactive, but both internal and terminal ramets could die independently from each other, with a probability of *d*_i_ for internal ramets, and with a probability of *d*_t_ for terminal ramets. When a ramet died, its connections to other ramets were deleted. If that ramet was in an internal position, the clone got fragmented.

States of the lattice cells were updated once in every ramet generation. The order of updating was basipetal: spacer tips were updated first in random order; this was followed by updating the terminal ramets and afterwards the internal ramets, both also in random order.

We investigated different growth forms varying the mortality of internal ramets (*d*_i_) and the mortality of terminal ramets (*d*_t_) in the range of 0.01 to 0.5. We used the following values: *d*_i_, *d*_t_ ∈ {0.01, 0.02, 0.03, 0.04, 0.05, 0.10, 0.15, 0.20, 0.25, 0.30, 0.35, 0.40, 0.45, 0.50}.

We defined non-steady-state and quasi-steady-state cases and determined the number of steps which were suitable for making a reliable difference between these two types of cases. We used this given number of steps for testing the proportion of quasi-steady-state cases (*Q*) in 1000 repetitions and the efficiency of occupation (i.e. ramet numbers) (*N*) within quasi-steady-state cases. Data analysis was performed using the R for Windows version 3.4.3 environment, in RStudio version 1.1.419 (RStudio Team 2015).

## Results

### Non-steady-state and quasi-steady-state cases

**Fig. 2:**
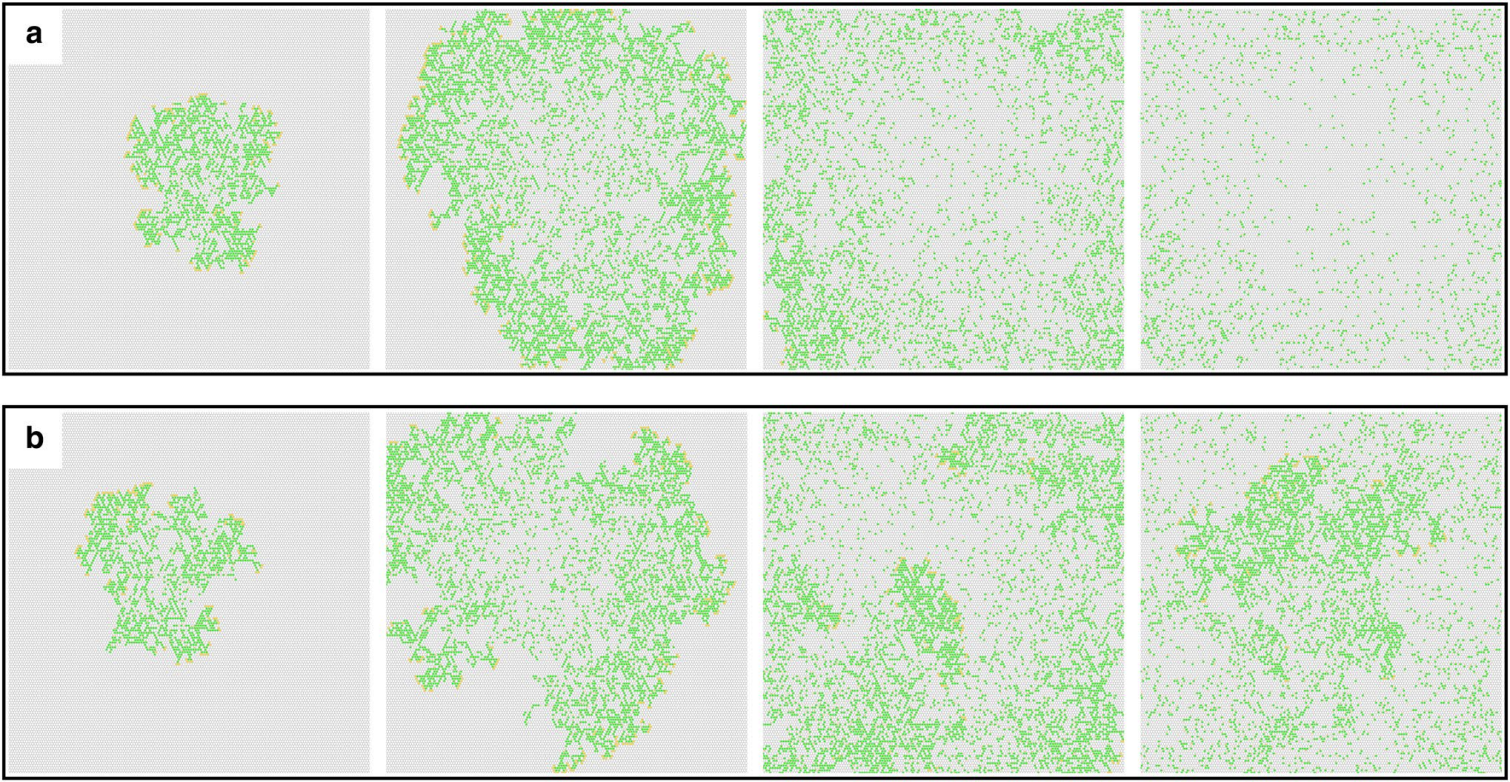
Examples for non-steady-state and quasi-steady-state cases at *d*_i_= 0.01, *d*_t_= 0.30. **a** Emergence of a non-steady-state, **b** emergence of a quasi-steady-state at about the 100th, 200th, 300th and 400th steps

We detected two main types of outcomes in our model: non-steady-state and quasi-steady-state cases. In non-steady-state cases, all terminal ramets died before the plant was able to spread in the field or when the spreading front reached the boundaries of the field. In the case of absence of terminal ramets, the plant stopped growing, and its internal ramets died back (Fig. 2a). In quasi-steady-state cases, the plant was able to spread in the field and could recolonize areas that became empty in the meantime (Fig. 2b).

We found that 1000 was the suitable number of steps to allow reliable differentiation between the two types of cases.

**Fig. 3:**
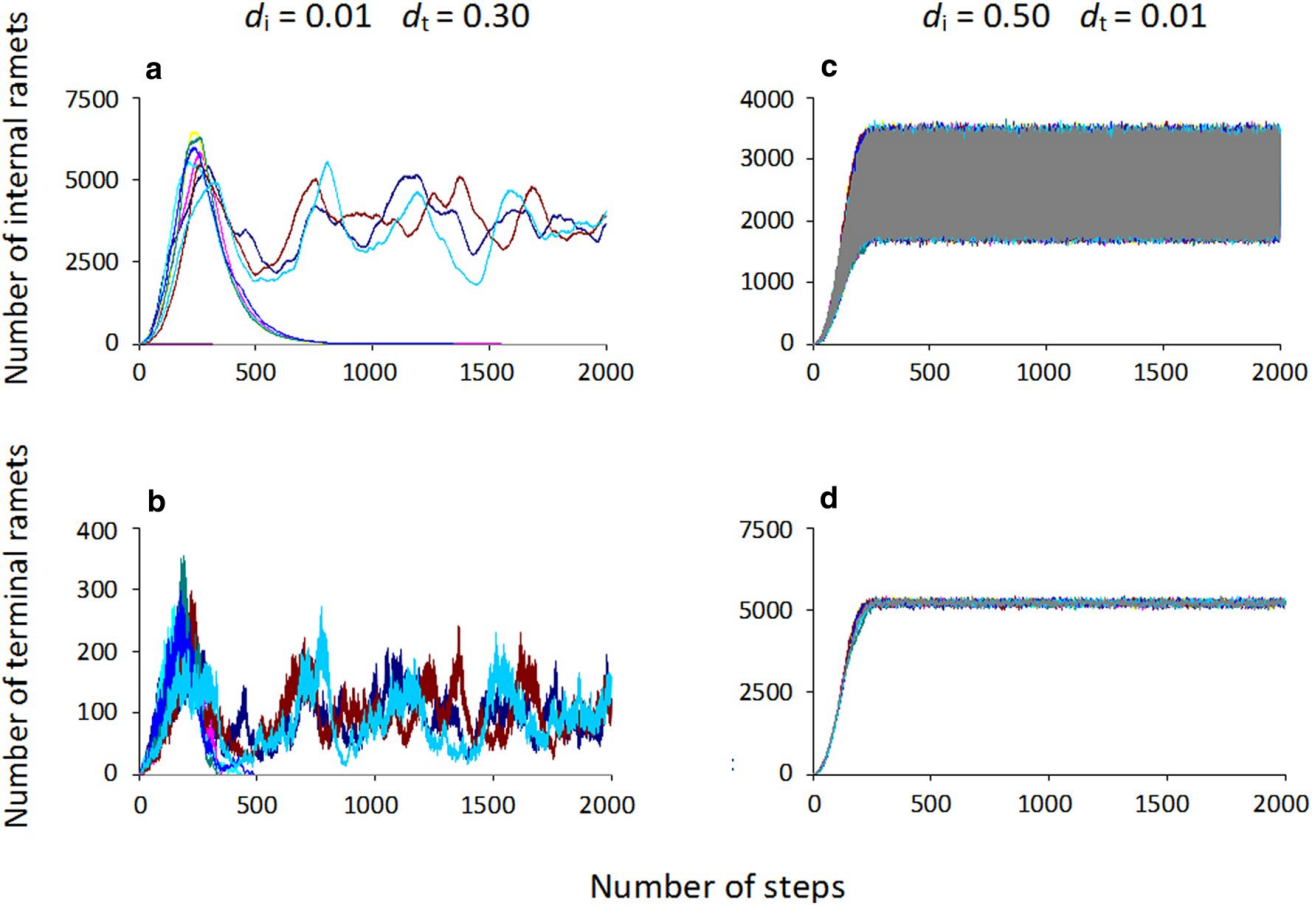
Time dependence of **a** internal ramet numbers and **b** terminal ramet numbers at low internal ramet mortality and relatively high terminal ramet mortality. **c** Time dependence of internal and **d** terminal ramet numbers at high internal and low terminal ramet mortality

In non-steady-state cases at relatively high terminal ramet mortality (*d*_t_), the number of terminal ramets decreased to zero in the first few steps where internal ramet mortality (*d*_i_) was not very low, or in about 500 steps where *d*_i_ was very low (Fig. 3b). In these cases, the growth of the plant stopped and internal ramets died back (Fig. 3a). In quasi-steady-state cases, there were terminal ramets present at the 1000th step.

**Fig. 4:**
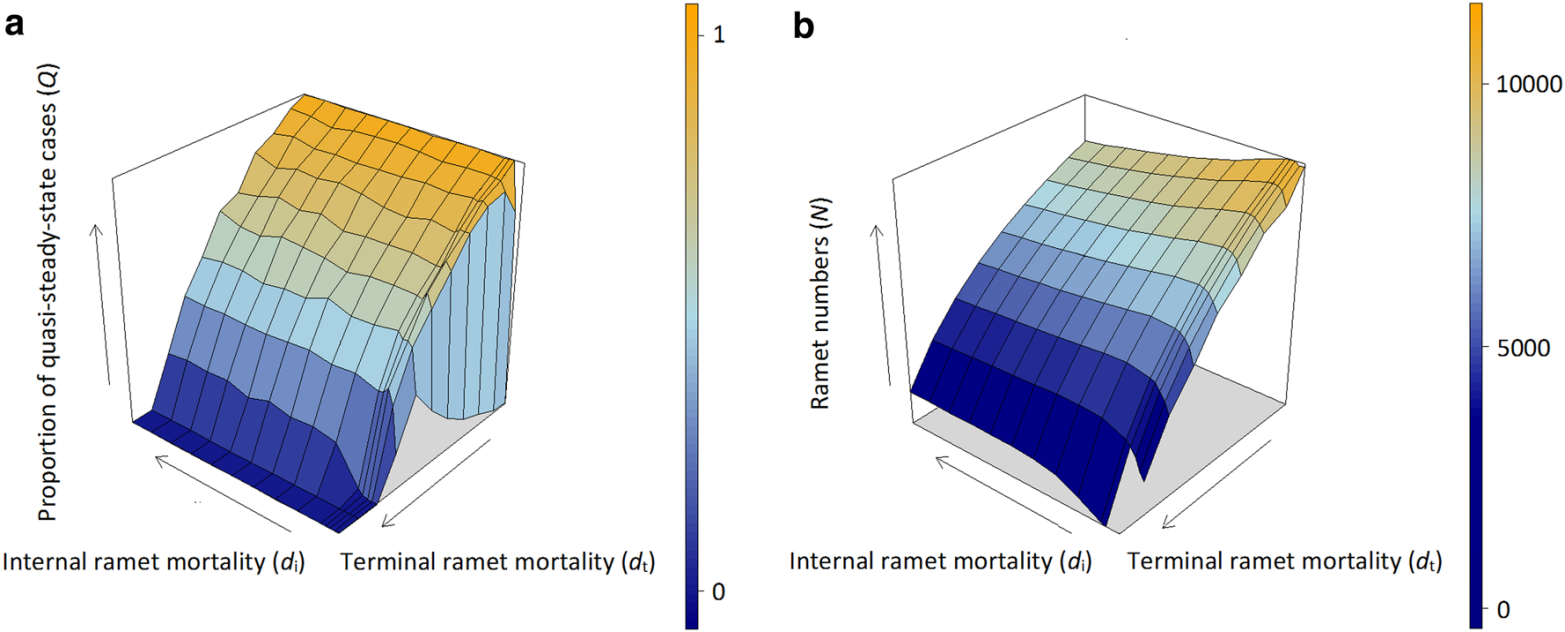
**a** Proportion of quasi-steady-cases in 1000 repetitions as a function of internal and terminal ramet mortality, **b** efficiency of occupation in quasi-steady-state cases as a function of internal and terminal ramet mortality

For those parameter combinations where *d*_t_ was low and *d*_i_ was not very low, non-steady-state cases were rare and the plant usually died back in the first few steps (Figs. 3c, d, 4a).

For those parameter combinations where *d*_t_ was low and *d*_i_ was very low, quasi-steady-state cases were very rare (Fig. 4a)–usually there were no terminal ramets (i.e. quasi-steady-state cases) at the 1000th step.

### Proportion of quasi-steady-state cases and efficiency of occupation

At very low values of *d*_i_ and *d*_t_, the proportion of quasi-steady-state cases (*Q*) was very low. When increasing *d*_i_, we experienced a sharp increase in *Q.* However, when increasing *d*_i_ further, *Q* remained approximately constant. (The extreme value *d*_i_ = 1 would mean that the plant consists only of terminal ramets.)

At very low values of *d*_i_ with increasing *d*_t_, we experienced an increase followed by a decrease in *Q.* At higher values of *d*_i_, increasing *d*_t_ resulted in a decrease in *Q.* At *d*_t_= 0.5, *Q* was equal to zero for all values of *d*_i_ (Fig. 4a).

The efficiency of occupation (i.e. ramet numbers) (*N*) tended to decrease with increasing *d*_i_ and *d*_t_, but not the lowest ramet mortality resulted in the highest value of *N*. Very low values of *d*_i_ also resulted in a decrease in *N* (Fig. 4b).

### The spreading front

**Fig. 5:**
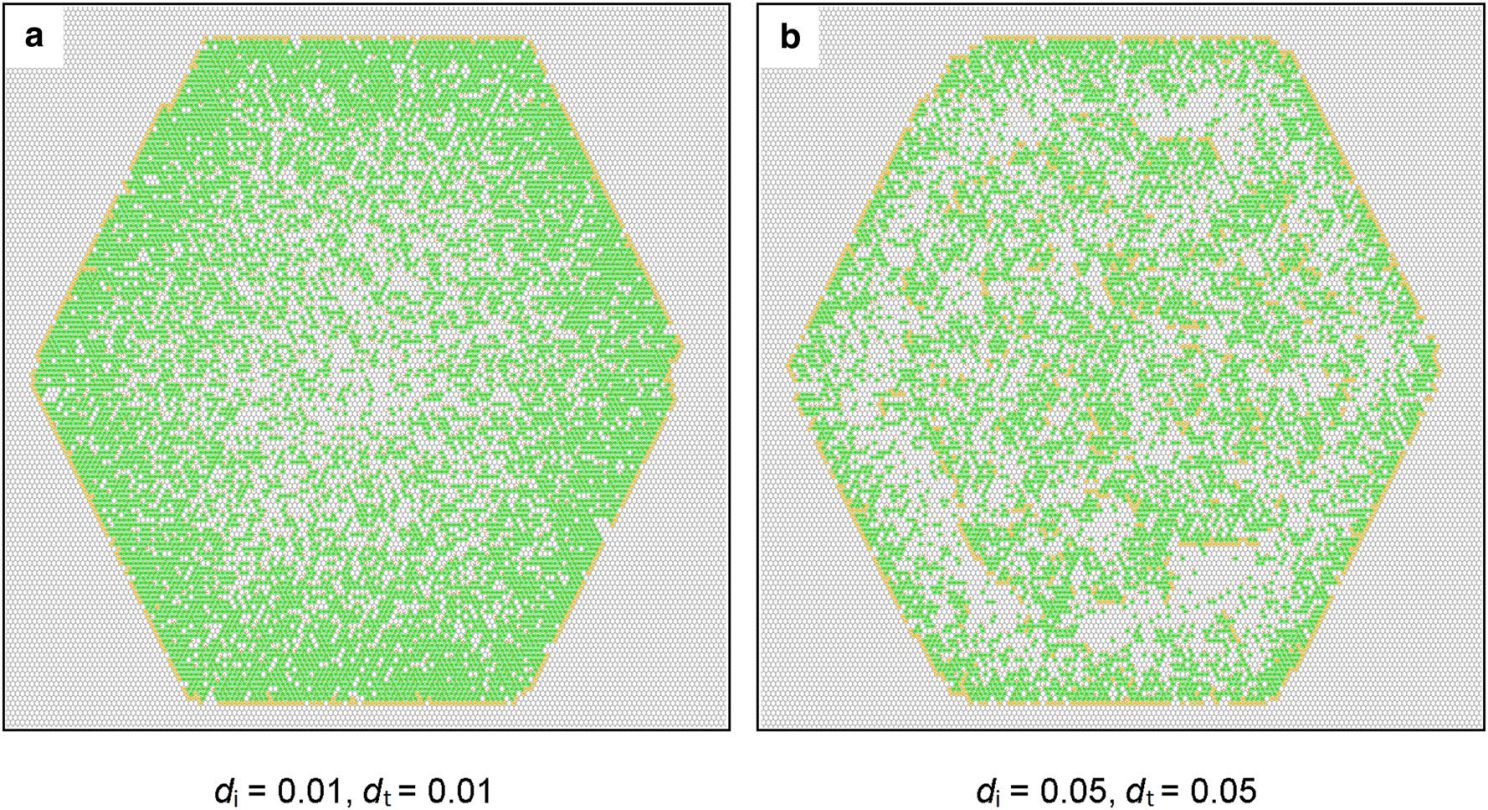
**a** The spreading front with very low ramet mortality, **b** the spreading front with higher ramet mortality

At very low values of *d*_i_ and *d*_t_, the spreading front and the areas behind were dense (Fig. 5a), associated with low values of *Q* (Fig. 4a). With the increase in *d*_i_ and *d*_t_, these areas became less dense (Fig. 5b). This phenomenon allowed the spreading front to return and thus recolonize the vacated sites in the central area, resulting in an increase in *Q* (Figs. 4b, 5b).

## Discussion

Our expectation was that higher ramet mortality would be associated with lower occupation. Our results only partially verified this hypothesis. Although the average ramet numbers tended to decrease with increasing ramet mortality, it was not the lowest ramet mortality that caused the highest occupation. In case of very low ramet mortality rates, the spreading front and the area behind this front were so packed that the plant was not able to return and recolonize the vacated sites in the central area. This led towards a lower proportion of quasi-steady-state cases (*Q*) and lower occupation in these cases.

The increase in *d*_i_ resulted in a sharp increase in *Q*, as the area behind the spreading front became less dense and allowed the recolonization of the vacated sites in the central area. Further increase in *d*_i_ did not result in further increase in *Q.*

The increase in *d*_t_ resulted in an increase followed by a decrease in *Q* at very low values of *d*_i_. The explanation for the initial increase in *Q* is that the spreading front and the area right behind this front, which was very dense at very low value of *d*_i_, became less dense. However, in general, the increase in *d*_t_ is unfavourable for the plant. The increase in *d*_t_ at higher levels of *d*_i_ (i.e. less dense area behind the spreading front) resulted in a monotonous decrease in *Q.* At *d*_t_= 0.5 the plant dies back from a single terminal ramet statistically in 50% of the cases. *Q* was equal to zero at this extremely high value of *d*_t_ with all values of *d*_i_.

In a field study, Pitelka et al. (1985) found that ramet mortality was size dependent (the larger ramets being more likely to survive). They reported that mean mortality rates were generally low (3.9–6.1%). Our results are consistent with this empirical study, as we have shown that a low (but not too low) level of internal ramet mortality is required for continued survival of the genet. In our study, every terminal ramet tried to develop offspring into adjacent cells (i.e. spacers were short); it leads to a packed spreading front. A suitable level of ramet mortality was required for the plant to return and recolonize the central area of the field. A study by Tamm et al. (2002) showed a positive correlation between ramet longevity and vegetative mobility (i.e. the distance between a parent and its offspring ramet). They discussed that it leads to a higher turnover rate of ramets and may possibly contribute to the higher potential species richness of the affected community. Considering the above, we suppose that ramet mortality has a great influence not only on intraclonal considerations, but also on the organization of plant communities. In a research conducted by Adachi et al. (1996), the development of *Reynoutria japonica* Houttuyn was studied with a stochastic computer simulation model. This species is a pioneer clonal herb in a volcanic desert of Japan, forming extended circular monoclonal patches. As a patch develops, central die-back occurs, i.e. shoot density of the plant decreases in the centre of the patch. The results of the model showed that the central die-back is caused by the plant itself via its internal rule of branching (acute branching angles). Establishment of secondary successional species occurs only in these parts of the patches. We found central die-back without recolonization associated with very low ramet mortality, which was not sufficient for opening the spreading front. In our recent study, the branching angle was always acute. In one of our further studies, we tested the effects of branching angle on clonal growth. We found fan-shaped growth pattern at acute branching angle and more circular pattern for the growth form where both acute and wide angles could occur. The growth form with acute branching angle occupied more space (i.e. had relatively more ramets) in most of the simulated habitats. However, the effect was weak in general. The only exception occurred at extremely high ramet mortality (*d*_i_ and *d*_t_> 0.8), when the ramet density behind the spreading front was low, and thus, immediate backtracking became feasible (Benedek et al. 2016). In this recent study, we did not use these extremely high ramet mortality values, as for these, the proportion of quasi-steady-state cases was zero in the homogeneous environment.

Although *Reynoutria* in the example above is an important successional species in Japan, it is a widespread invasive plant in Europe and North America (e.g. Maurel et al. 2010). The ability of clonal growth may have an important role in some exotic species invading seminatural vegetation (Vogt-Andersen 1995); thus, studying clonal growth is also important from the aspect of conservational biology. To eliminate or control invasive species, we need to understand their behaviour. Our results may contribute to a deeper understanding of clonal plant growth and its limiting factors (e.g. ramet longevity or other rules internal to the plant).

In this research, we only varied the internal and terminal ramet mortality and studied the clonal plant growth in a homogeneous environment. Presumably, other growth rules internal to the plant, e.g. the most basic architectural rules: branching angle, probability of branching and internode length (see in e.g. Herben and Suzuki 2002) and environmental heterogeneity, also had an influence on our results. We tested the effects of ramet mortality on clonal plant growth using some of these parameter combinations too, and we found that our results are valid in a considerable range of the parameter space (data not shown). Our further studies tested clonal plant growth also in a heterogeneous environment (Benedek et al. 2016; Oborny et al. 2017), and we varied the scale of habitat patchiness compared to the scale of growth (Oborny et al. 2017). However, without field studies, these do not yet provide a full picture of environmental controls and growth rules internal to the plant. A comprehensive study of this issue would be an interesting and important subject for future research (see a pioneering study by Wildová et al. 2007).
